# COVID-19 in Kidney Transplant Recipients Vaccinated With Oxford–AstraZeneca COVID-19 Vaccine (Covishield): A Single-center Experience From India

**DOI:** 10.1097/TP.0000000000003835

**Published:** 2021-05-26

**Authors:** Hari Shankar Meshram, Vivek B. Kute, Nauka Shah, Sanshriti Chauhan, Vijay V. Navadiya, Ansy H. Patel, Himanshu V. Patel, Divyesh Engineer, Subho Banerjee, Jamal Rizvi, Vineet V. Mishra

**Affiliations:** 1 Department of Nephrology and Transplantation Sciences, Institute of Kidney Diseases and Research Center, Dr HL Trivedi Institute of Transplantation Sciences (IKDRC-ITS), Ahmedabad, Gujarat, India.; 2 Department of Medicine, BJ Medical College, Ahmedabad, Gujarat, India.; 3 Department of Transplantation Surgery, IKDRC-ITS, Ahmedabad, Gujarat, India.; 4 Department of Gynecology, Institute of Kidney Diseases and Research Center, Dr HL Trivedi Institute of Transplantation Sciences (IKDRC-ITS), Ahmedabad, Gujarat, India.

## Abstract

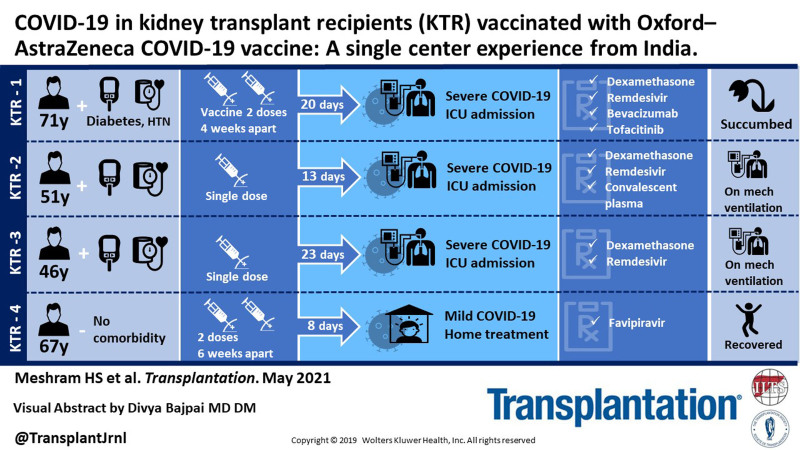

Severe acute respiratory syndrome coronavirus (SARS-CoV2) has been reporting global peaks in India in April 2021. The enormous task of vaccinating such a bulk population has become more difficult in the state where health resources for managing coronavirus disease 2019 (COVID-19) are rapidly emptying amid the COVID-19 surge. Indian advisories have approved 2 vaccines: Oxford–AstraZeneca COVID-19 (Covishield) and BBV152 (Covaxin) as of April 2021. The efficacy of these vaccines has been reported lesser compared with the mRNA vaccines in general population.^1,2^ Preliminary reports with mRNA vaccine in organ transplant patients have shown that around 75% of transplant patients have a suboptimal response to mRNA COVID-19 vaccine.^3^ Such data with Covishield vaccine are lacking. We report 4 cases of COVID-19 in kidney transplant recipients (KTRs) 2 of them after 2 doses of Covishield vaccine and another 2 after a single dose. The study is approved by the Institutional Ethics committee. We also abided by the Declaration of Helsinki and Declaration of Istanbul principles. None of them reported any minor or major side effects postvaccination. The probable exposure was community acquired in all the 4 cases. Three patients were on triple immunosuppression. Three cases required critical care admission and received steroids, remdesivir, anticoagulation, and investigational therapies (patient 1: tofacitinib and bevacizumab; patient 2: COVID-19 convalescent plasma) as anti-COVID-19 therapy during the stay. Patient 1 died and patients 2 and 3 are on mechanical ventilation with a poor prognosis (Table 1). Patient 4 had a mild COVID-19 course being managed at home. Time from the second dose of vaccine to onset of symptoms was 20 and 8 d for patients 1 and 4, respectively. Patients 2 and 3 developed symptoms after 13 and 23 d of the first dose of vaccine, respectively. SARS-CoV2 IgG antibody response was suboptimal in 3 cases, while seroconversion developed in patient 1. To the best of our knowledge, this is the first report of COVID-19 postvaccination after Covishield vaccine in KTRs. The documentation of attenuated response in our report bolsters our speculation that antibody response after Covishield might be suboptimal in KTRs. The recent reports^3^ of insufficient antibody response from mRNA vaccine whose efficacy is around 95% in general population compared with the lower efficacy vaccine used in Indian settings is alarming for the immunosuppressed group as they will have further attenuated response. The outcome of vaccinated patients acquiring COVID-19 is sparsely reported. There have been a few reports of COVID-19 infection postvaccination in organ transplants after mRNA vaccines.^4,5^ In conclusion, critical COVID-19 despite vaccination in our report is concerning and it emphasizes the fact that KTRs are more prone to COVID-19 even after vaccination. Hence, safety measures to prevent disease transmission should be continued. There is a special need to find a definitive therapy for COVID-19, as reports of vaccine efficacy in transplants are worrisome. The future implications of our report highlight the need for further research with reporting of outcome of vaccinated patients, efficacy of different vaccines, dosages, schedules, seroprotection levels, and antibody durability in KTRs.

**TABLE 1.** Summary of the case developing COVID-19 after 2 doses of Oxford–AstraZeneca COVID-19 vaccine

	**1**	**2**	**3**	**4**
Demographic characteristics				
Age (y)	71	51	46	67
Sex	Male	Male	Male	Male
Comorbidities	Hypertension, diabetes	Hypertension, diabetes	Hypertension, diabetes	None
Basic disease	Diabetic nephropathy	ADPKD	Hypertension	ADPKD
Transplant	Living-related transplant	Deceased donor	Living-related transplant	Deceased donor
Induction	No induction	Thymoglobulin	No induction	Thymoglobulin
Ant-rejection therapy	No	Yes (AMR [ag1 PTC score 1 C4d2] in immediate transplant)	Yes (chronic rejection)	No
Time from transplant to COVID-19	16 y	1.5 y	9 y	6 y
Baseline serum creatinine(mg/dL)	1.4	1.8	4.32	1.5
Oxford–AstraZeneca vaccination schedule	4 wk apart; 2 doses	1 dose	1 dose	6 wk apart; 2 doses
Onset of symptom after vaccine	20 d	13	23	8 d
COVID-19 exposure	Community	Community	Community	Community
Molecular analysis of SARS-CoV2 RT-PCR test	Positive	Positive	Positive	Positive
ORF fab, CT value	29	16	–	15
E gene, CT value	22	–	22	–
N gene, CT value	24	16	24	–
SARS-CoV2 seroconversion (Yes/No)	Yes	No	No	No
Clinical symptoms on admission				
Fever	8 d	1 d	1 d	–
Cough	8 d	1 d	1 d	1 d
Difficulty of breathing	5 d	–	1 d	–
Weakness	–	–	1 d	–
Diarrhea	–	1 d	–	–
Vitals on presentation				
Pulse (per min)	110	98	102	84
Respiratory rate (per min)	16	18	18	16
Temperature (Fahrenheit)	98.6	100.2 F	98.6	98.6
Systolic blood pressure (mm Hg)	152	130	142	130
Diastolic blood pressure (mm Hg)	104	90	86	80
Oxygen requirement on admission	Nonrebreather mask	Low-flow oxygen	Nonrebreather mask	Ambient air
Eastern Cooperative Oncology Group score on admission	Capable of only limited self-care	Capable of only limited self-care	Capable of only limited self-care	Fully active, able to carry on predisease performances without restriction
Acid–base gas analysis				Not done
pH	7.23	7.23	7.2	–
Pco_2_ (mm Hg)	53	30	34	–
Po_2_ (mm Hg)	64	74	72	–
HCO_3_^–^ (mEq/L)	19	12.5	12	–
Base deficit	5.8	13	14	–
Laboratory abnormalities on admission (normal range)				
Hemoglobin (13.6–16 g/dL)	12	11	7.9	10.9
RBC count (4.6–6.2 million/mm^3^)	4.6	3.6	4	4.13
TLC (4–11 × 1000/mm^3^)	15.45	4.5	15.9	4.8
Platelet count (150–400 × 1000/mm^3^)	340	177	216	214
Neutrophils (60%–70%)	90	88	93	88
Lymphocytes (25%–33%)	8	10	5	9
Eosinophils (2%–6%)	1	1	1	1
Monocytes (4%–7%)	1	1	1	2
PCV (42%–52%)	40	33.9	26.2	33.4
MCV (82–92 FL)	88	92	81.9	80.9
MCH (27–32 pg)	27	29	24.7	26.4
MCHC (32–36 g/dL)	31	32	30.2	32.6
RDW (10.6%–15.7%)	14	13.5	15.3	15.5
aPTT (28–35 s)	64.2	–	–	32
PT-INR (0.64–1.8)	1.7	–	–	1.03
D-dimer (200–500 ng/mL)	370	1010	1230	330
ALP (64–306 IU/L)	58	54	56	40
Total bilirubin (0.3–1.2 mg/dL)	0.8	0.6	0.4	0.6
AST (0–40 IU/L)	52	17	25	22
ALT (0–40 IU/L)	46	24	58	20
Total protein (6–8.3 g/dL)	6.4	5.2	6.5	6.5
Albumin (3.2–5 g/dL)	6.43.5	3.2	3.4	3.5
Globulin (2.5–3.5 g/dL)	2.9	2	3.10	2
Blood urea	45	90	90	42
Serum creatinine (0.5–1.4 mg/dL)	1.45	2.75	Hemodialysis dependent	1.52
Serum chloride (96–108 mEq/L)	100	98	102	101
Serum sodium (135–145 mEq/L)	139	131	141	136
Serum potassium (3.5–4.5 mEq/L)	4.4	3.7	4.2	4.12
IL-6 (<7 pg/mL)	119	179	24	–
hs-CRP (0–10 mg/L)	198	51.2	46	2.0
PCT (<0.5 ng/mL)	0.09	0.44	0.69	
HCV ELISA	Nonreactive	Nonreactive	Nonreactive	Nonreactive
HIV ELISA	Nonreactive	Nonreactive	Nonreactive	Nonreactive
HBsAg ELISA	Nonreactive	Nonreactive	Nonreactive	Nonreactive
Serum ferritin (13–400 ng/mL)	364	1000	–	124
Baseline medicines				
Immunosuppression				
Tablet prednisolone	5 mg OD	10 mg OD	7.5 mg OD	10 mg OD
Tablet cyclosporine	50 mg BD	–		–
Tablet azathioprine	–	–	–	75 mg OD
Capsule tacrolimus	–	1.5 mg -1 mg	–	1.25 mg -1 mg
Tablet mycophenolate	750 mg BD	360 mg TDS	360 mg TDS	–
Antihypertensive				
Tablet clonidine			0.1 mg TDS	
Tablet nifedipine			20 mg TDS	
Tablet metaprolol	–	50 mg OD	–	25 mg OD
Tablet diltiazem	–	–	–	30 mg BD
Tablet cilnidipine	5 mg OD	–	–	–
Tablet telmisartan	–	–	40 mg OD	–
Tablet losartan	50 mg BD	–	–	–
Antidiabetic		–	–	–
Injection human Mixtard (50/50)	32 IU morning	–	–	–
Injection human Mixtard (30/70)	16 IU night	–	–	–
Injection basalog	–	6 IU	–	–
Tab vildagliptin			50 mg BD	–
Tablet gliclazide	30 mg OD	–	-	–
Tablet metformin	1000 mg BD	–	-	–
Others				
Tablet clopidogrel	75 mg OD	–	–	–
Tablet atorvastatin	10 mg OD	–	–	–
Tablet tamsulosin	0.4 mg OD	0.4 mg OD	–	–
Treatment regimen for COVID-19 received during stay	Critical care admission	Critical care admission	Critical care admission	Home-based therapy
Immunosuppression	CNI + antimetabolite stopped	CNI + antimetabolite stopped	CNI + antimetabolite stopped	No change
Tablet azithromycin	–	500 mg OD	500 mg OD	500 mg OD
Tab favipiravir	–	–	–	1600 mg BD; 800 mg BD for 5 d
Tablet tofacitinib	10 mg BD	–		–
Injection dexamethasone, 6 mg OD since admission	Yes	Yes	Yes	–
Remdesivir 200 mg on d 1, followed by 100 mg for 5 d	Yes	Yes	Yes	–
Injection bevacizumab	400 mg on d 4 of admission	–	–	–
COVID-19 convalescent plasma component	–	2 doses	–	–
Injection LMWH (0.6 S/C OD)	Yes	Yes	Yes	–
BiPAP	Since 12 h of admission	On d 7 of admission	Since d 2 of admission	–
Mechanical ventilation	On d 4 of admission	On d 14 of admission	–	–
Outcome	Died on d 8 of admission	Admitted on ventilator, d 16 of admission	Admitted on ventilator, post-CPR on d 4 of admission, dialysis dependent	At home, stable and recovered

ADPKD, autosomal polycystic kidney disease; ALP, alkaline phosphatase; ALT, alanine transaminase; aPTT, activated partial thromboplastin time; AST, alanine transferase; BiPAP, bi-level positive pressure ventilation; CNI, calcineurin inhibitors; COVID-19, coronavirus disease; ELISA, enzyme-linked immunosorbent assay; HBsAg, hepatitis B virus; HCO_3_-, bicarbonate; HCV, hepatitis C virus; hs-CRP, high sensitive C-reactive protein; IL-6, interleukin-6; LMWH, low-molecular-weight heparin; MCH, mean corpuscular hemoglobin; MCHC, mean corpuscular hemoglobin concentration; MCV, mean corpuscular volume; PCT, procalcitonin; PCV, packed cell volume; PT-INR, prothrombin time – international normalized ratio; RBC, red blood cell count; RDW, red cell distribution width; SARS-CoV2 RT-PCR, severe acute respiratory syndrome coronavirus real rime polymerase chain test; TLC, total leukocyte count.

## ACKNOWLEDGMENTS

The authors express our sincere gratitude to all the resident doctors and healthcare staffs who are tirelessly doing a mammoth job of managing the COVID-19 cases in India, despite facing resource crisis.
